# Spontaneous mutations in *CYC8* and *MIG1* suppress the short chronological lifespan of budding yeast lacking SNF1/AMPK

**DOI:** 10.15698/mic2018.05.630

**Published:** 2018-02-19

**Authors:** Nazif Maqani, Ryan D. Fine, Mehreen Shahid, Mingguang Li, Elisa Enriquez-Hesles, Jeffrey S. Smith

**Affiliations:** 1 Department of Biochemistry and Molecular Genetics, University of Virginia School of Medicine, Charlottesville, VA 22908.; 2 Department of Laboratory Medicine, Jilin Medical University, Jilin, 132013, China.

**Keywords:** Snf1, AMPK, Cyc8, Cat8, aging, yeast, chronological lifespan, TPR

## Abstract

Chronologically aging yeast cells are prone to adaptive regrowth, whereby mutants with a survival advantage spontaneously appear and re-enter the cell cycle in stationary phase cultures. Adaptive regrowth is especially noticeable with short-lived strains, including those defective for SNF1, the homolog of mammalian AMP-activated protein kinase (AMPK). SNF1 becomes active in response to multiple environmental stresses that occur in chronologically aging cells, including glucose depletion and oxidative stress. SNF1 is also required for the extension of chronological lifespan (CLS) by caloric restriction (CR) as defined as limiting glucose at the time of culture inoculation. To identify specific downstream SNF1 targets responsible for CLS extension during CR, we screened for adaptive regrowth mutants that restore chronological longevity to a short-lived *snf1*∆ parental strain. Whole genome sequencing of the adapted mutants revealed missense mutations in TPR motifs 9 and 10 of the transcriptional co-repressor Cyc8 that specifically mediate repression through the transcriptional repressor Mig1. Another mutation occurred in *MIG1* itself, thus implicating the activation of Mig1-repressed genes as a key function of SNF1 in maintaining CLS. Consistent with this conclusion, the *cyc8* TPR mutations partially restored growth on alternative carbon sources and significantly extended CLS compared to the *snf1*∆ parent. Furthermore, *cyc8* TPR mutations reactivated multiple Mig1-repressed genes, including the transcription factor gene *CAT8*, which is responsible for activating genes of the glyoxylate and gluconeogenesis pathways. Deleting *CAT8* completely blocked CLS extension by the *cyc8* TPR mutations on CLS, identifying these pathways as key Snf1-regulated CLS determinants.

## INTRODUCTION

The budding yeast SNF1 complex is homologous to metazoan AMP-activated protein kinase (AMPK), and acts as a sensor of cellular energy status that adjusts metabolism in response to environmental nutrient conditions and stress 1,2,3. Like AMPK, SNF1 is a heterotrimer consisting of a catalytic α-subunit (Snf1), an activating γ-subunit (Snf4), and one of three possible β-subunits (Gal83, Sip1, Sip2) that tether Snf1 and Snf4 together and contribute to cellular localization and substrate specificity for the kinase activity 3,4. Snf1 is activated by low glucose concentrations through phosphorylation of its activation loop at threonine 210 by one of three upstream kinases (Sak1, Tos3, Elm1) 5. Snf4 regulates accessibility of phosphorylated T210 to the phosphatase Glc7, such that under low glucose conditions, ADP association with the Snf4 γ-subunit protects Snf1 T210 from dephosphorylation 6,7.

*Saccharomyces cerevisiae* is a facultative anaerobe that prefers to utilize glycolysis and fermentation for producing ATP and carbon skeletons, rather than the mitochondrial TCA cycle and oxidative phosphorylation, to support rapid cell proliferation under aerobic conditions. SNF1 alters metabolism in response to low environmental glucose in part by phosphorylating metabolic enzymes such as acetyl CoA carboxylase (ACC). ACC converts acetyl CoA into malonyl CoA, which is the rate-limiting step of *de novo* fatty acid biosynthesis 8. Snf1 phosphorylation of Acc1 inactivates the enzyme, thus promoting fatty acid catabolism and accumulation of acetyl CoA, which then contributes to histone acetylation and transcriptional activity stimulated by low glucose 9. Snf1 also functions more directly in transcriptional activation by phosphorylating several transcription factors (Mig1, Cat8 and others) that are responsible for diminishing glycolysis/fermentation pathways and promoting the utilization of alternative carbon sources such as ethanol and acetate 2. This transition in transcription and metabolism is known as the diauxic shift, and is important for long-term cell survival in stationary phase cultures 10.

The period of yeast cell survival during stationary phase is also known as chronological lifespan (CLS), which is commonly used as a simple model for aging of non-mitotic cells 11. CLS is typically measured by assaying cell viability in liquid cultures either by the ability to form colonies on fresh media or using viability dyes such as FUN-1 and propidium iodide 12. Numerous genetic modifications and environmental conditions impact CLS. For example, CLS is extended by inhibiting the TOR signaling pathway 13, which is also observed for metazoan models of aging ranging from *C. elegans* to mice 14. CLS is also significantly extended by reducing glucose concentration at the time of culture inoculation from 2% (non-restricted/NR) to 0.5% (calorie restricted/CR) 15. CR in this context has a significant impact on gene expression and cellular physiology during the diauxic shift and into stationary phase 16. CR also prevents the accumulation of organic acids like acetic acid in the growth medium 17,18, which can have detrimental effects on cell viability during chronological aging 17.

One of the mechanisms by which CR prevents acetic acid accumulation is by promoting acetate consumption, as evidenced by genes involved in alternative carbon source utilization being among the most abundant gene class upregulated by CR in aging cells 16. Consistent with ethanol and acetate consumption, CR prevents acetyl CoA depletion of cells in stationary phase cultures, most likely due to the combined action of Snf1 in phosphorylating ACC and in preventing inactivation of acetyl CoA synthetase genes *ACS1* and *ACS2* during the diauxic shift and into stationary phase 19. While SNF1 is best known for its role in derepressing genes in the absence of glucose, including genes involved in fatty acid oxidation and utilization of non-fermentable carbon sources 20, it also regulates the cellular responses to oxidative stress 21 and DNA damage 22,23, all of which can impact yeast chronological lifespan 24. SNF1 is critical for maintaining CLS and is required for the extension of CLS induced by CR growth conditions 18,19,25, raising the question of which downstream SNF1 targets or cellular processes are most critical for cell survival, and which could also have important implications for cell survival mechanisms regulated by AMPK. To identify critical aging-relevant downstream targets of Snf1, we screened for adaptive regrowth from chronologically aging cultures of *snf1*∆ cells and utilized whole genome DNA sequencing of the adapted isolates to identify any mutated genes. Remarkably, almost every isolate had a mutation in specific TPR (tetratricopeptide repeat) motifs of the transcriptional co-repressor *CYC8* that mediate Mig1-dependent repression of genes normally activated by SNF1 during the diauxic shift. RNA-seq and qRT-PCR revealed at least partial transcriptional restoration to a subset of such genes with Mig1 binding sites, including *CAT8*, a transcriptional activator of glyoxylate and gluconeogenesis pathway genes. *CAT8* was also required for the restoration of longevity in adapted isolates, thus implicating transcriptional derepression of *CAT8* as a key SNF1 function in maintaining CLS.

## RESULTS

**Figure 1:**
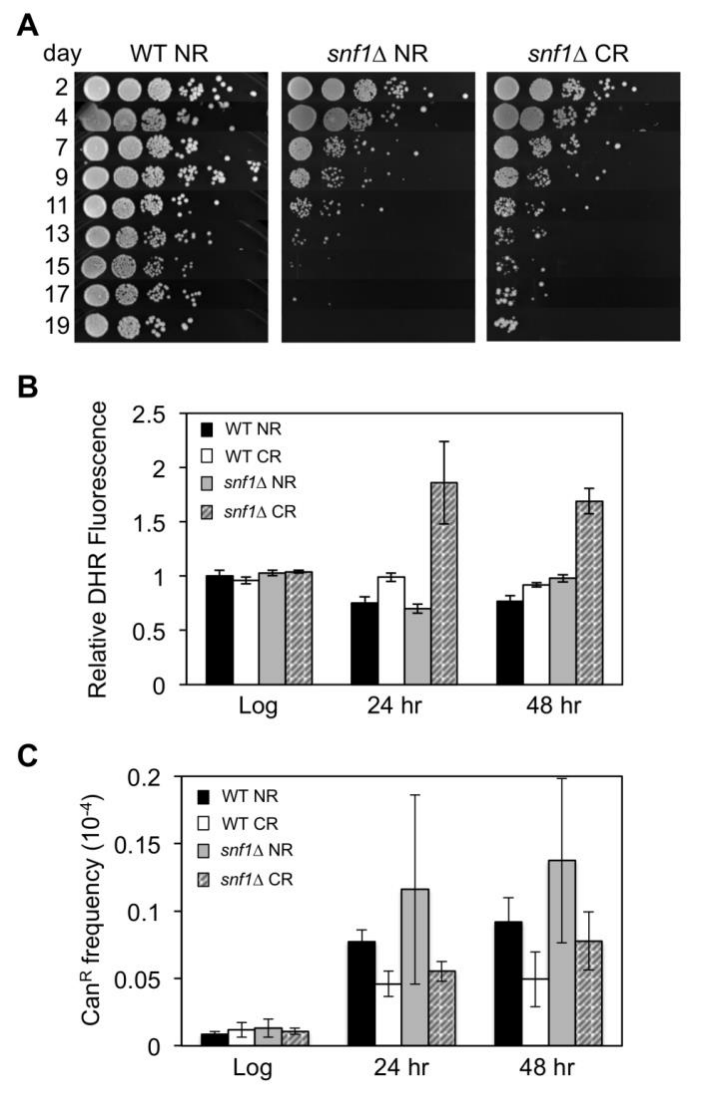
FIGURE 1: Frequent adaptive regrowth of *snf1*∆ strains during chronological aging under caloric restriction (CR- 0.5% glucose) conditions. **(A)** Qualitative CLS assay showing the rapid loss of viability by a *snf1*∆ strain (JS1394) compared to WT (JS1256) as the cells chronologically age in expired SC NR (2% glucose) medium. **(B)** Reactive oxygen species (ROS) accumulation in *snf1*∆ strains grown into stationary phase during CR culture conditions, as indicated by dihydrorhodamine 123 (DHR) staining. **(C)** *CAN1* gene mutation frequency in WT and *snf1*∆ strains grown under NR and CR conditions.

Cells deleted for the *SNF1* gene have a very short CLS under both NR and CR growth conditions 19. However, we often observe a phenomenon known as adaptive regrowth or "gasping" in *snf1*∆ cultures, especially under CR. When this happens, cell viability drops rapidly during the first 11 days for the *snf1*∆ mutant, but then eventually stabilizes or even recovers thereafter (Fig. 1A), as cells within the population are able to re-enter the cell cycle and slowly proliferate. Adaptive regrowth was previously shown to occur through uncharacterized genetic mutations that presumably allow utilization of nutrients released into the medium by dying cells 26. Since SNF1 targets multiple transcription factors, metabolic enzymes, and even histone H3 27, we hypothesized that mutations in genes allowing *snf1*∆ cells to survive in stationary phase during chronological aging would reveal critical targets or cellular processes that Snf1 regulates to promote CLS.

Yeast cultures under CR conditions (0.5% glucose) tend to accumulate reactive oxygen species (ROS) in the form of H_2_O_2_ during chronological aging 28. This results in a hormetic response, which activates superoxide dismutases that contribute to enhanced cell viability. One possible reason for the frequent adaptive regrowth of *snf1*∆ strains could therefore be elevated ROS levels that drive mutagenesis. Indeed, cellular staining with dihydrorhodamine (DHR) detected significantly elevated ROS levels in the calorie restricted *snf1*∆ mutant post-log phase after 24 or 48 hr of growth (Fig. 1B). However, mutation frequency at the *CAN1* gene was not significantly higher in the *snf1*∆ mutant compared to WT (Fig. 1C). It is therefore possible that high ROS levels in the *snf1*∆ mutant contribute to DNA damage that is effectively repaired, but the CR condition provides a strong selective pressure or ideal growth condition for specific classes of mutants to adapt.

### A genetic screen for *snf1*∆ CLS bypass suppressor mutations

To identify *snf1*∆ bypass suppressors, we took advantage of the frequent adaptive regrowth observed for the *snf1*∆ strain as observed in Fig. 1A. Single colonies of the *snf1*∆ strain JS1394 were purified and independently inoculated into 10 different SC (0.5% glucose) cultures (A1-A10), which were then chronologically aged. 10-fold serial dilutions were spotted onto rich YPD agar plates every 2 or 3 days to monitor for adaptive regrowth, which was easily noticeable as sustained and enlarged colony growth after approximately 11 days (Fig. 1A, CR culture). Putative adapted mutants were chosen from the YPD plates as they appeared starting at day 11, with three candidates picked and single colony purified from culture A1. Over subsequent days, 2 candidate mutants were picked from most of the other cultures (up to day 17) for a total of 20 candidates (Table 1). Only 1 candidate was isolated from culture A7.

**Table 1:** Mutations identified from *snf1*∆ bypass suppressor CLS screen. ^a ^Amino acid substitutions. ^b ^Recessive (R) or semi-dominant (SD) growth phenotype on YEP+glycerol/ethanol plates. ^c ^Mutation in the promoter. ^d ^Frameshift mutation at A352 that creates a stop codon at codon 357.

Mutant	Strain	ORF	Gene name	Chr	Mutation	Change^a^	Dom^b^
A1-1	JS1413	*YBR112C*	*CYC8*	II	464597 C/G	A392P	R
		*YHR118C*	*ORC6*	VIII	344796 C/G	C278S	
		*YMR218C*	*TRS130*	XIII	706605 C/G	V95L	
A1-2	JS1414	*YBR112C*	*CYC8*	II	464597 C/G	A392P	R
		*YMR218C*	*TRS130*	XIII	706605 C/G	V95L	
A1-3	JS1415	*YBR112C*	*CYC8*	II	464597 C/G	A392P	R
		*YMR218C*	*TRS130*	XIII	706605 C/G	V95L	
A2-1	JS1416	*YBR112C *	*CYC8*	II	464762 C/T	G337S	SD
A2-2	JS1418	*YBR112C *	*CYC8*	II	464662 A/C	L370R	R
		*YDR325W *	*YCG1*	IV	1119362 C/T	A746V	
A3-1	JS1422	*YBR112C *	*CYC8*	II	464654 G/A	L373F	R
		*YPL212C*	*PUS1*	XVI	152608 C/G	C181S	
A3-2	JS1423	*YBR112C*	*CYC8*	II	464654 G/A	L373F	R
		*YPL212C*	*PUS1*	XVI	152608 C/G	C181S	
		*YBR275C *	*RIF1*	II	755428 T/C	N560S	
A4-1	JS1424	*YBR112C *	*CYC8*	II	464588 C/A	D395Y	SD
		*YMR153W *	*NUP53*	XIII	564421 C/T	-14^c^	
A4-2	JS1425	*YBR112C *	* CYC8*	II	464588 C/A	D395Y	R
		*YMR153W*	*NUP53*	XIII	564421 C/T	-14^c^	
		*YBR098W*	*MMS4*	II	443160 G/A	G549E	
A5-1	JS1426	*YBR112C*	*CYC8*	II	464723 A/C	L350V	SD
		* YBR112C*	*CYC8*	II	464715 ∆C	A352^d^	
A5-2	JS1427	*YBR112C*	* CYC8*	II	464723 A/C	L350V	R
		*YBR112C*	*CYC8*	II	464715 ∆C	A352^d^	
A6-1	JS1417	* YBR112C*	*CYC8*	II	464670 C/A	W367C	R
A6-2	JS1419	*YBR112C*	*CYC8*	II	464670 C/A	W367C	R
A7-1	JS1430	*YGL035C*	*MIG1*	VII	432935 C/A	C43F	SD
A8-2	JS1421	*YBR112C*	*CYC8*	II	464660 C/T	G371S	SD
		*YJL129C*	*TRK1*	X	174668 T/C	D880G	
A9-1	JS1428	*YBR112C*	*CYC8*	II	464660 C/T	G371S	R
A9-2	JS1429	*YBR112C*	*CYC8*	II	464660 C/T	G371S	R
		*YIL015W*	*BAR1*	IX	323228 C/G	P296R	
A10-1	JS1431	*YBR112C*	*CYC8*	II	464671 C/A	W367L	R
A10-2	JS1432	*YBR112C *	* CYC8*	II	464671 C/A	W367L	R
		*YBR135W*	* CKS1*	II	504917 T/G	L22V	
		*YJR080C*	*AIM24*	X	581354 G/A	S88F	

**Figure 2:**
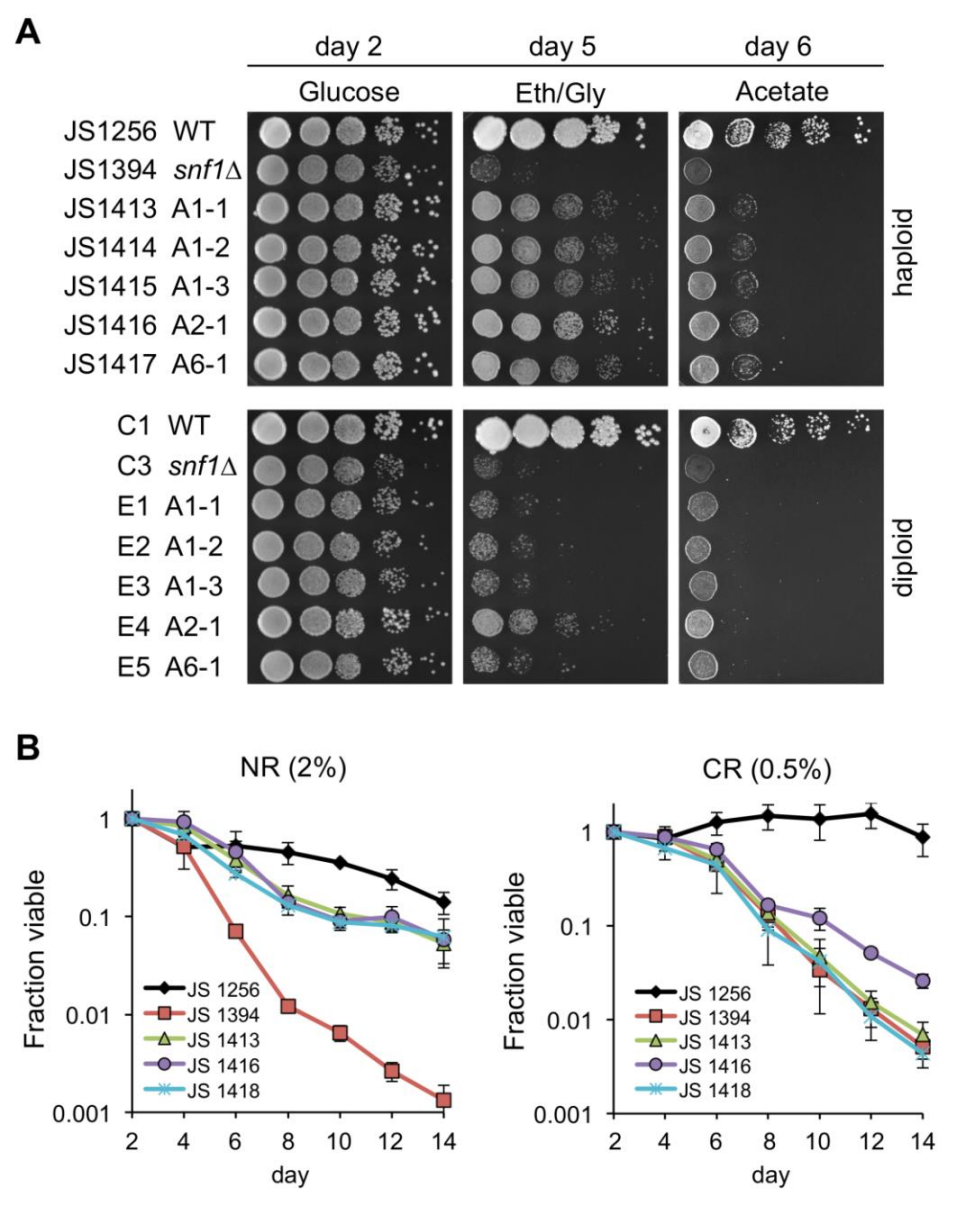
FIGURE 2: Characterization of *snf1*∆ bypass suppressor mutants that partially restore growth on alternative carbon sources and chronological lifespan. **(A)** Spot test growth assays of WT, *snf1*∆, and a subset of suppressor candidates on YEP plates containing glucose, ethanol/glycerol (Eth/Gly), or acetate as carbon sources. The top panel represents the haploid strains and the bottom panel represents a dominance test where the indicated *MAT*a haploid candidate (i.e. A1-1) was crossed to a *MAT*α *snf1*∆ strain (JS1399) to generate a diploid homozygous for *snf1*∆ and heterozygous for other mutations. **(B)** Quantitative CLS assays for WT (JS1256), *snf1*∆ (JS1394), and three suppressor candidates, JS1413, JS1416 and JS1418. Assays were terminated after 14 days to avoid any adaptive regrowth. Error bars indicate standard deviations.

Cells lacking SNF1 are unable to grow on alternative carbon sources such as glycerol, ethanol, or acetate 29. To test if the CLS-adapted *snf1*∆ mutants restored this abil-ity, 10-fold serial dilutions of each strain were spotted onto rich yeast extract/peptone (YEP) plates containing glucose, ethanol/glycerol, or acetate. As shown in Fig. 2A, the *snf1*∆ parental strain (JS1394) grew poorly on ethanol/glycerol and acetate plates, as expected. Each adapted mutant showed improved growth on both types of plates, suggesting they gained one or more secondary mutations that partially restored alternative carbon source utilization. We next used these growth phenotypes to test if the putative mutations were recessive or dominant by crossing each adapted strain to a *snf1*∆ mutant of the opposite (*MAT*α) mating type, thus generating homozygous *snf1*∆*/snf1*∆ diploids that were heterozygous for any other mutated loci. The restoration of growth on ethanol/glycerol and acetate plates observed with haploids was lost from most of the diploids, indicative of recessive alleles (Fig. 2A and Table 1). However, several diploids such as E4 did grow modestly better than the *snf1*∆*/snf1*∆ control diploid (C3), so they were considered semi-dominant. Next, several candidates from the screen were quantitatively retested for CLS under NR and CR conditions (Fig. 2B). The CLS of each candidate was longer than the *snf1*∆ parent (JS1394), but not as long as WT (JS1256). This partial recovery of CLS was similar to the partial growth phenotype observed on alternative carbon sources (Fig. 2A). CR slightly increased CLS of the *snf1*∆ parent, as previously described 19, but shortened CLS of two adapted mutants (JS1413, JS1418), as compared to NR (Fig. 2B). CLS of the semi-dominant A2-1 (JS1416) mutant was unaffected by CR. Importantly, CR did not extend CLS for any of the adapted mutants tested, suggesting that CR partially works through whichever functional gene(s) were mutated.

Next, genomic DNA was prepared from each adapted mutant, as well as the *snf1*∆ parent (JS1394), and Illumina DNA sequencing libraries were constructed using an NEBNext Ultra II DNA Library Prep Kit (New England Biolabs). Libraries were multiplexed and sequenced on an Illumina MiSeq apparatus, then aligned to the *S. cerevisiae* genome. SNPs specific to the adapted mutants with maximum quality scores and within open reading frames or promoters were identified and listed in Table 1. Mutations found in more than one isolate from a particular culture (i.e. A1-1, A1-2, and A1-3) were considered to be derived from the same progenitor cell. Taking this into account, a total of 23 unique mutations were recovered from 19 sequenced candidates, with the majority (15) occurring at cytosines. Consistent with mutagenesis caused by high ROS levels, oxidation products of cytosine such as 5-hydroxycytosine and 5-hydroxyuracil commonly result in C to T transitions and C to G transversions 30, which were the two most abundant mutation classes from the screen.

**Figure 3:**
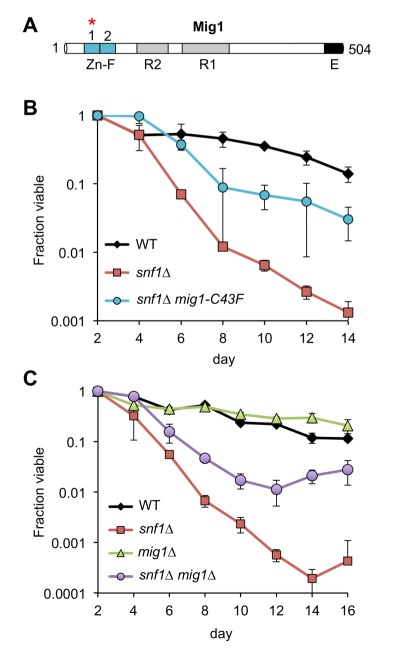
FIGURE 3: Mutations in *MIG1* partially restore CLS to a *snf1*∆ strain. **(A)** Diagram of Mig1 protein domain organization showing the two zinc fingers (Zn-F), two regulatory domains (R1 and R2) and an effector domain (E) 36. Red asterisk indicates the zinc finger mutated (C43F) in suppressor candidate JS1430. **(B)** Quantitative CLS assay showing short CLS of *snf1*∆ (JS1394) compared to WT (JS1256). The *snf1*∆* mig1-C43F* mutant (JS1430) extends CLS compared to *snf1*∆. **(C)** Quantitative CLS assay showing similar partial CLS extension by deleting *MIG1* in the *snf1*∆ background (JS1446). Deletion of *MIG1* had little CLS effect on its own (JS1442). CLS assays were performed in SC 2% glucose conditions. Error bars indicate standard deviation.

Remarkably, every adapted mutant other than A7-1 (JS1430) had a mutation in the *CYC8* gene, which encodes a subunit of the Cyc8/Tup1 corepressor complex that transcriptionally represses a large number of genes controlling mating-type, cell cycle, glucose repression, and others 31. *CYC8* was also the only mutated gene in 5 of the adapted mutants, clearly implicating *CYC8* as functionally relevant to the *snf1*∆ suppression phenotype. Furthermore, the only mutation in A7-1 caused a C43F amino acid substitution in Mig1, a transcriptional repressor that physically interacts with Cyc8/Tup1 at specific gene promoters under glucose repression and is a direct phosphorylation target of SNF1 32. Mig1 is a zinc finger protein that binds to 5’-MCCCCRS-3’ motifs of certain genes under glucose repression 33,34. Cys43 is located in the first of two C2H2 zinc finger motifs that mediate DNA binding by Mig1 (Fig. 3A), so the C43F mutation in A7-1 (JS1430) is predicted to generally disrupt Mig1 function by preventing association with target gene promoters. If this were true, then simply deleting *MIG1* should produce the same *snf1*∆ suppression CLS phenotype as C43F. As shown in Fig. 3B, the short CLS of a *snf1*∆ mutant was extended by *mig1-C43F*. Similarly, deleting *MIG1* also partially suppressed the short *snf1*∆ CLS phenotype (Fig. 3C), thus implicating transcriptional release of Mig1 target gene repression as a critical Snf1 function for maintaining CLS. Since Snf1 phosphorylation of Mig1 normally activates Mig1-repressed genes under low glucose conditions such as the diauxic shift period 35, we hypothesized that impairing Mig1 and/or Cyc8 function was facilitating transcription of such genes in the absence of Snf1, thus bypassing the *snf1*∆ mutation.

### Mutations in specific TPR motifs of *CYC8* restore CLS to cells lacking *SNF1*

**Figure 4:**
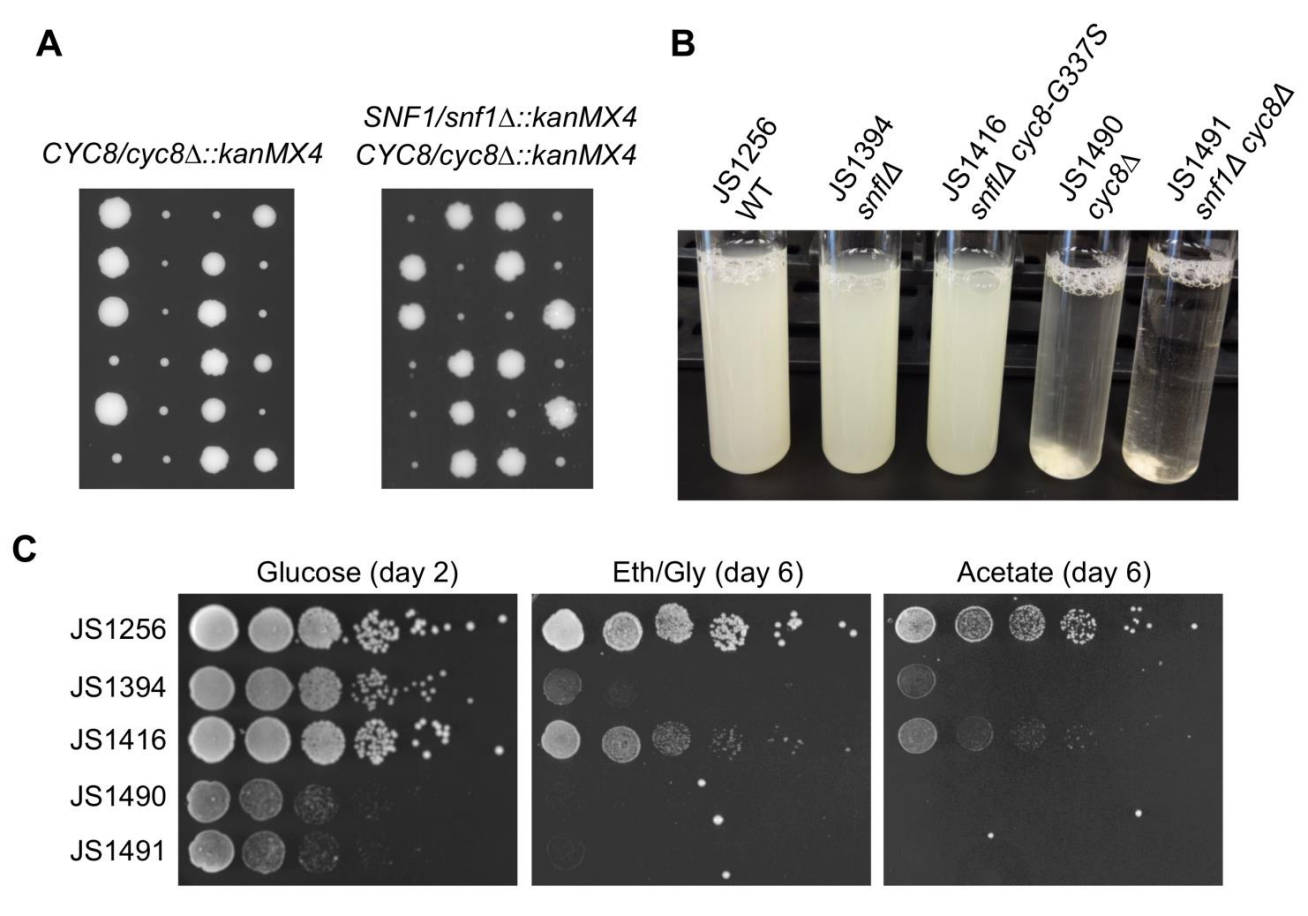
FIGURE 4: Distinct phenotypic differences between adaptation-derived *cyc8* mutants and *cyc8*∆ mutants. **(A)** Growth of spores from tetrad dissection of *CYC8/cyc8*∆*::kanMX4* or *SNF1/snf1*∆*::kanMX4* *CYC8/cyc8*∆*::kanMX4* heterozygous diploids. Spores were dissected onto YPD plates and grown for 5 days. **(B)** Flocculence phenotype of strains deleted for *CYC8* compared to a representative adapted mutants from the screen (*cyc8-G337S*). Cultures were grown for 2 days on a roller drum, then briefly vortexed and allowed to settle for 5 min. **(C)** Spot test growth assay comparison of *snf1*∆ (JS1416) suppression phenotype compared to the *snf1*∆* cyc8*∆ mutant (JS1491), which grows very poorly. A *cyc8*∆ mutant (JS1490) also grows poorly.

*SNF1* (sucrose non-fermenting) was originally described as a mutation that prevents growth on sucrose due to an inability to express *SUC2*, a glucose-repressed gene encoding the sucrose hydrolyzing enzyme invertase 37. Another name for *CYC8* is *SSN6*, which stands for suppressor of snf1 38. Mutations in *CYC8* were identified as *cyc1-13* revertants that restore growth on lactate due to increased expression of cytochrome C 39, while mutations in *SSN6* were identified as revertants that allow *snf1* mutants to grow on sucrose 38. The *cyc8* and *ssn6 *mutations turned out to be allelic and were eventually shown to derepress transcription of glucose-repressed genes that require Snf1 for their activation under low glucose conditions. Several other *"SSN" *genes have been described, including several subunits of the Mediator complex 40, but only *cyc8/ssn6*

and *mig1/ssn1* mutations were identified from our screen for CLS extension of a *snf1*∆ mutant. Additionally, all but one of the *cyc8* mutations were missense amino acid substitutions, suggesting that complete elimination of Cyc8 protein could be detrimental. To determine if deleting *CYC8* would have the same suppression effect as observed with the *snf1*∆* mig1*∆ mutant (Fig. 3B), we generated *cyc8∆::kanMX snf1*∆*::kanMX* double mutants through genetic crossing and tetrad dissection. All resulting *cyc8*∆ spores grew poorly regardless of the accompanying *SNF1* allele (Fig. 4A), and were highly flocculent (Fig. 4B), consistent with previously described Cyc8/Tup1-dependent repression of flocculence genes such as *FLO1* 41. In stark contrast, the adapted *snf1*∆* cyc8* mutants all grew robustly (Fig 2A and 4B), and were highly dispersed in liquid culture, similar to WT and *snf1*∆ strains (Fig. 4B, JS1416 shown as example). Furthermore, *snf1*∆* cyc8*∆ strains did not grow at all with ethanol/glycerol or acetate provided as the carbon sources (Fig. 4C), implying that *cyc8* mutations isolated in the CLS screen were highly specialized.

**Figure 5:**
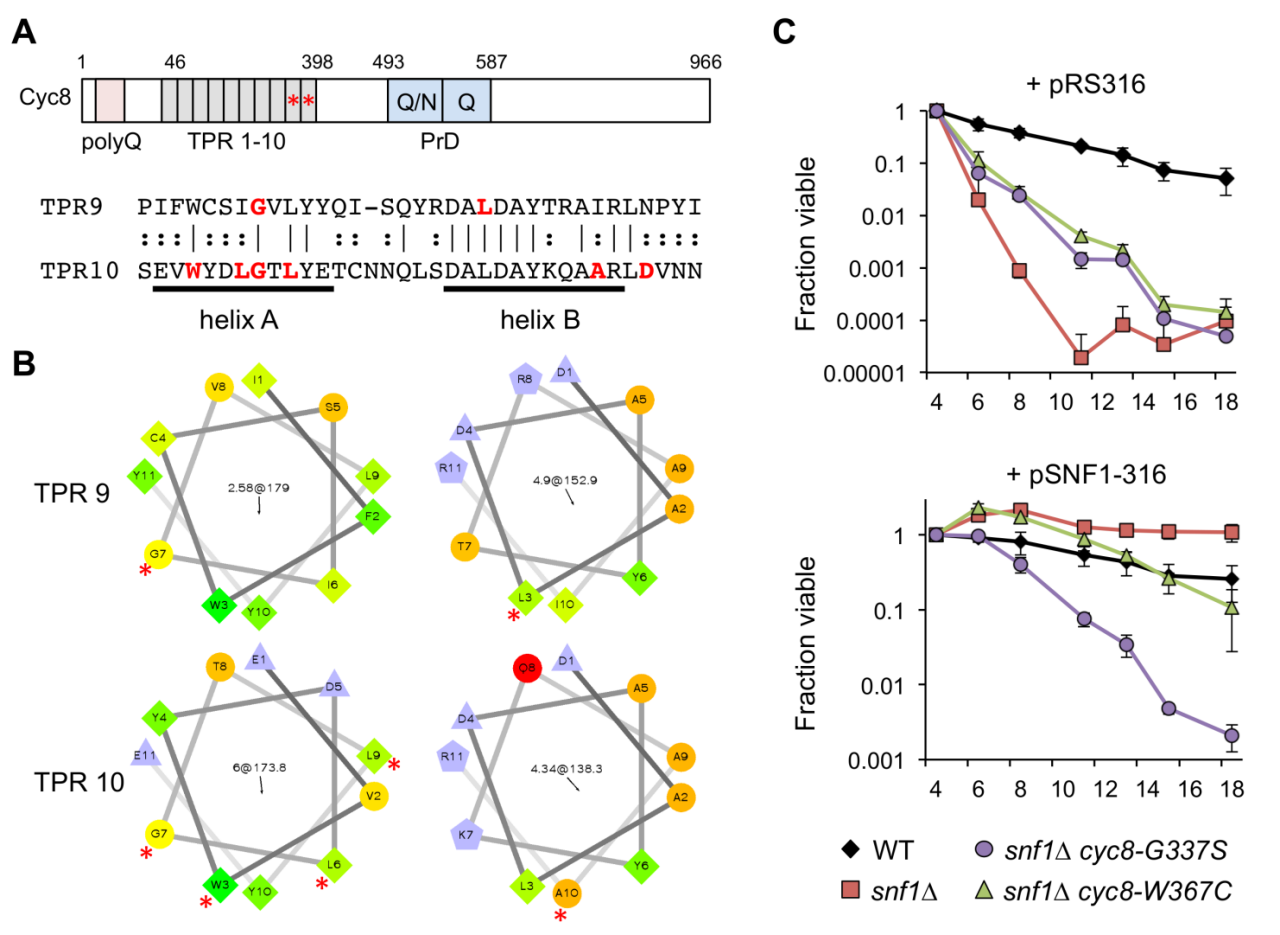
FIGURE 5: Adaptive Cyc8 mutations are specific to the TPR9 and TPR10 motifs. **(A)** Domain organization of the Cyc8 protein, indicating an N-terminal poly-glutamine (polyQ) tract, an array of 10 TPR motifs from amino acids 46 to 398, and the prion domain (PrD) consisting of polyQ/N and polyQ repeats. Asterisks indicate the location of mutations in the TPR9 and TPR10 motifs. An alignment of the TPR9 and TPR10 motifs is shown, indicating amino acids that were changed in the adapted strains. Note the mutations are distributed across the characteristic amphipathic α-helices. **(B)** Helical wheel representation of the A and B α-helices of TPR9 and TPR10 generated online (http://rzlab.ucr.edu/scripts/wheel/wheel.cgi . Mutated residues are indicated by an asterisk. **(C)** Quantitative CLS when SNF1 is restored to a *cyc8* TPR9 mutant (JS1416, *snf1*∆ *cyc8-G337S*) or a *cyc8* TPR10 mutant (JS1417, *snf1*∆* cyc8-W367C*) by transformation with a *CEN-ARS URA3* plasmid containing the *SNF1* gene (pSNF1-316). The empty *URA3* vector (pRS316) was also transformed as a control. Cells were maintained in SC-ura medium with 2% glucose throughout the experiment. Error bars indicate standard deviations.

Closer inspection of the *cyc8* mutations showed they all occurred in the 9^th^ or 10^th^ TPRs of an N-terminally located tandem array (Fig. 5A and Table 1). TPR motifs consist of two amphipathic α-helices (A and B) that mediate physical interactions with other proteins 42. All but one of the amino acids altered by missense mutation was hydrophobic and located within the predicted A and B α-helices (Fig. 5A). Furthermore, each altered amino acid was predicted by helical wheel projections to be on the hydrophobic face of either helix A or B (Fig. 5B), suggesting the mutations altered specific hydrophobic protein-protein interactions between Cyc8 and an associated factor. The most likely associated factor is Mig1 because the TPR8-10 motifs of Cyc8 mediate transcriptional repression by Mig1 43. There were no mutations identified within TPR8 from our screen, suggesting that TPR9 and TPR10 provide most of the function relevant to CLS.

Under activating growth conditions, the bulk of Mig1 is exported from the nucleus 44. However, significant levels of Mig1 and Cyc8/Tup1 remain associated with target gene promoters such as *GAL1 *and help recruit the SAGA co-activator complex 45, arguing that Cyc8/Tup1 is a transcriptional "co-regulator".rather than just a co-repressor. We therefore hypothesized that the TPR9 or TPR10 mutations in *CYC8* could potentially impact CLS independent of the *snf1*∆ mutation. To test this idea, *SNF1* was restored in a TPR9 mutant (JS1416) and TPR10 mutant (JS1417) by transformation with a *CEN/ARS* plasmid containing the *SNF1* gene (pSNF1-316), or an empty control plasmid (pRS316). Transformants were then quantitatively assayed for CLS in non-restricted (2% glucose) SC medium lacking uracil to maintain the plasmid. As shown in Fig. 5C, both mutants showed the expected partial CLS extension compared to *snf1*∆ when transformed with pRS316, recapitulating CLS suppression results from Fig. 2B. However, the TPR9 mutant (*snf1*∆* cyc8-G337S*) remained short lived when transformed with pSNF1-316, while CLS of the TPR10 mutant (*snf1*∆* cyc8-W367C*) was restored to normal (Fig. 5C). The *cyc8-G337S* mutation therefore impaired CLS in-dependently of *SNF1*, perhaps due to altered interactions with other transcription factors that associate with the TPR9 motif rather than TPR10. Such a model is also supported by the semi-dominant growth phenotype of this mutant on ethanol/glycerol or acetate media (Fig. 2A).

**Figure 6:**
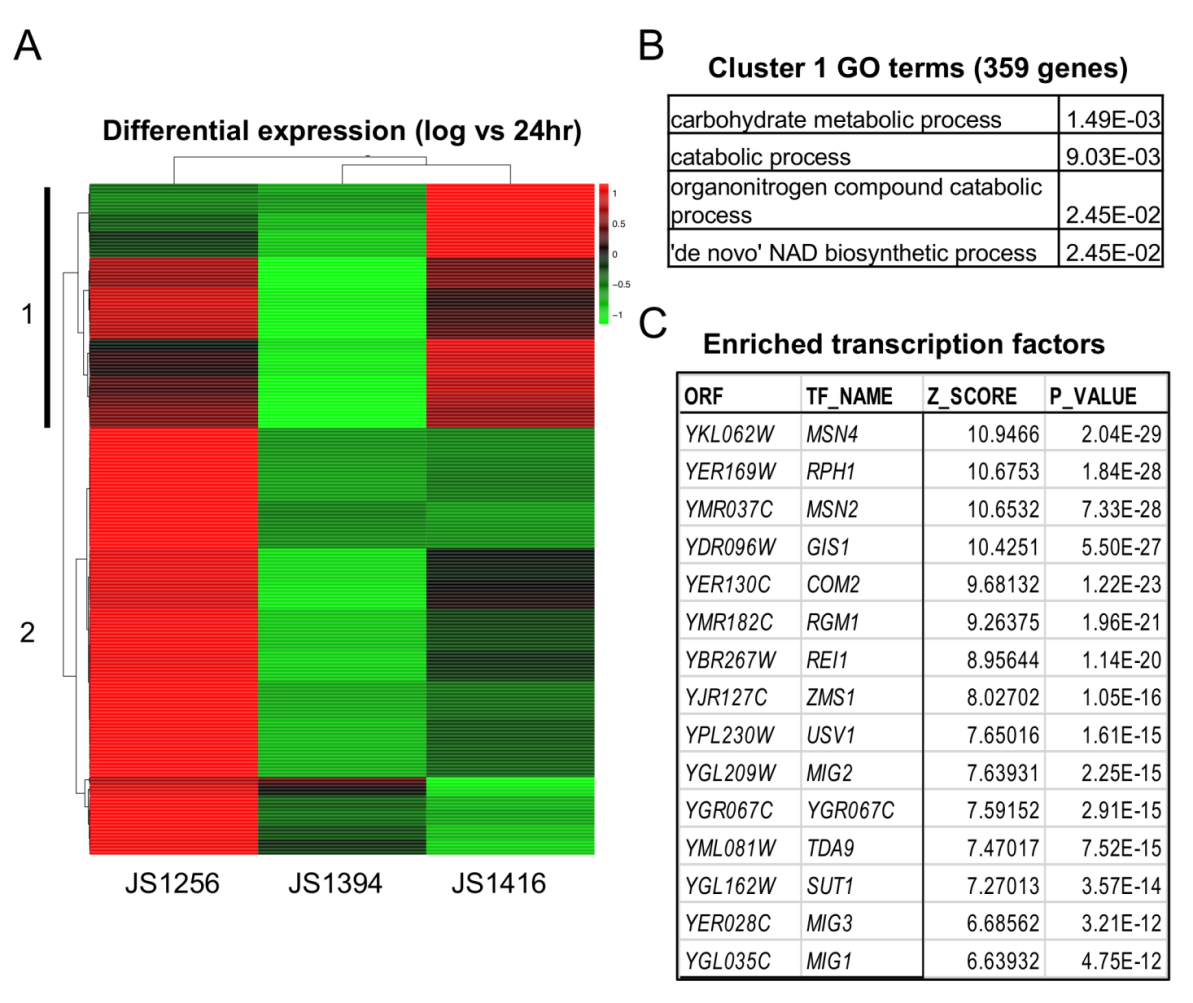
FIGURE 6: RNA-seq analysis of gene expression in WT, *snf1*∆, and bypass suppressor strains. **(A)** Genes (986) that were upregulated at 24 hr in the WT strain (JS1256), but not in the snf1∆ strain (JS1394) were analyzed by Pearson correlation clustering and displayed as a heat map of Z-score from the mean of all three strains. Cluster 1 (vertical black bar) represents 359 genes in which expression was restored in JS1416 compared to JS1394. **(B)** Gene ontology (GO) terms and p-values associated with cluster 1 using Holm-Bonferroni correction. **(C)** Top 15 enriched transcription factors on the promoters of genes with >2-fold enrichment in JS1416 compared to JS1394 (from Supplemental Table S1).

Next, RNA-seq analysis was performed on WT (JS1256),* snf1*∆ (JS1394) and the cyc8-G337S mutant (JS1416) that were grown for 6 (log), 24, or 48 hrs. Analysis focused on 986 genes that were upregulated at 24hr compared to log in the WT strain, but not in *snf1*∆. Unsupervised cluster analysis revealed a subset of 359 genes whose expression was at least partially restored in the suppressor strain (Fig. 6A). These genes were significantly enriched for catabolic processes and surprisingly, *de novo* NAD^+^ biosynthesis (Fig. 6B). As predicted, Mig1 binding sites were significantly enriched on the promoters of these genes, as were its related factors Mig2 and Mig3 (Fig. 6C), which appear to fine-tune glucose repression 46. However, among the top hits in P-value were four other transcription factors (Msn2/Msn4, Gis1 and Rph1) that are known multi-copy suppressors of *snf1*-defective phenotypes 47,48. Msn2/4 are major transcriptional activators for the general stress response 49, and Gis1/Rph1 are paralogous histone H3-K36 demethylases that can act as repressors or activators depending on the gene and the growth condition, including glycerol and acetate metabolism 2. Perhaps the *cyc8-G337S* mutation disrupts proper transcriptional regulation mediated by one or more of these, or other highly enriched factors listed in Fig. 6C and supplementary Table S3.

### Bypass suppression of *snf1*∆ by *cyc8-G337S* is *CAT8*-dependent

**Figure 7:**
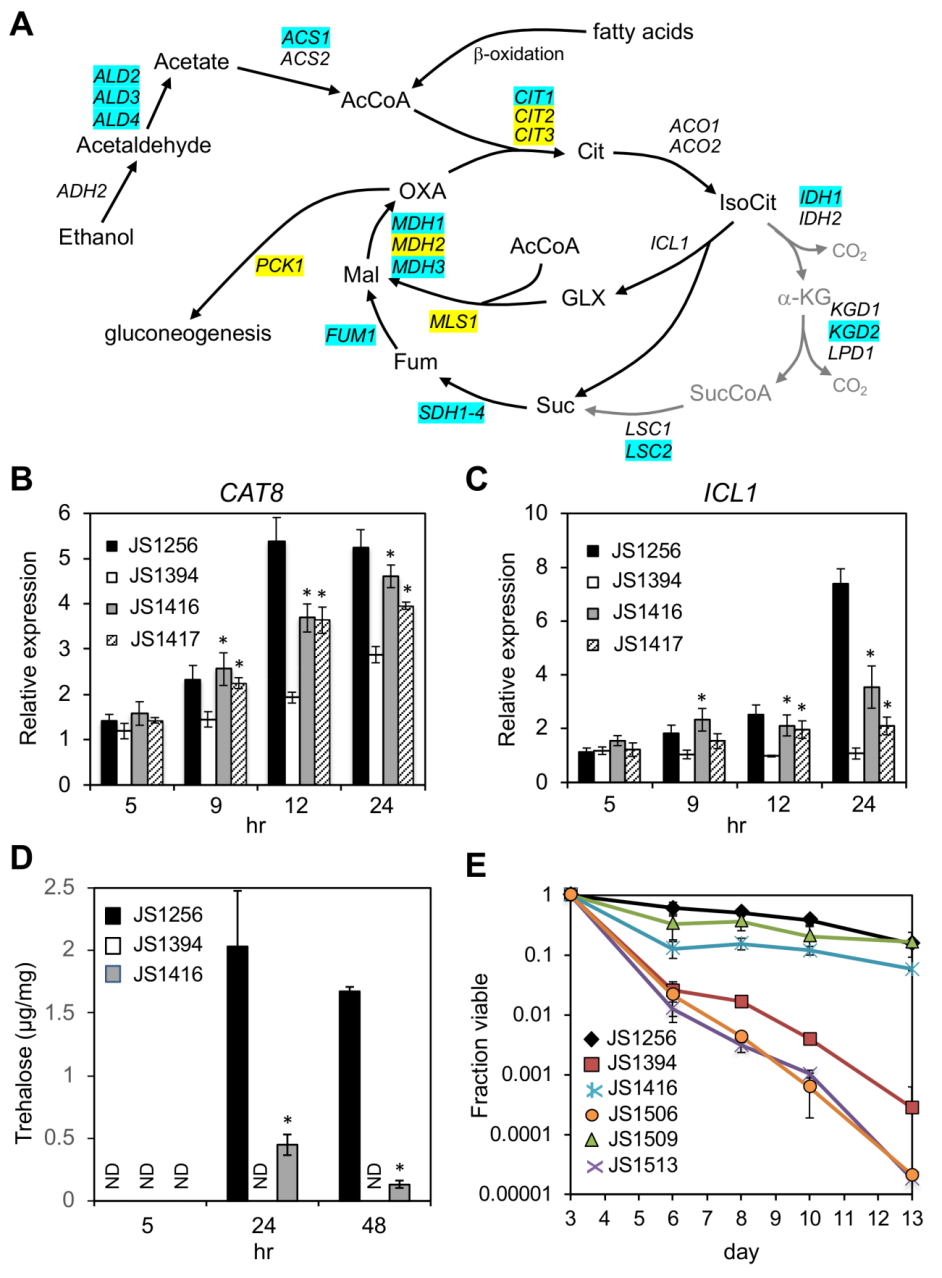
FIGURE 7: Reactivation of *CAT8* and the glyoxylate/gluconeogenesis pathways in adapted *snf1*∆ mutants. **(A)** Diagram of ethanol and fatty acid catabolism through the TCA and glyoxylate cycles, leading to gluconeogenesis. Genes involved in each enzymatic step are listed, including the first step of gluconeogenesis (*PCK1*). Blue shading indicates restored expression in the suppressor strain (JS1416) at 24 or 48 hr based on RNA-seq analysis. Yellow shading indicates restored expression only at 48 hr. Abbreviations: Cit, citrate; IsoCit, isocitrate; GLX, glyoxylate; Mal, malate; OXA, oxaloacetate; α-KG, α-ketoglutarate; SucCoA, succinyl CoA; Fum, fumarate. **(B)** Quantitative RT-PCR of *CAT8* expression when entering the diauxic shift. **(C)** Quantitative RT-PCR of *ICL1* expression during the same time course. **(D)** Trehalose measurements from WT (JS1256), *snf1*∆ (JS1394), and suppressor (JS1416) cells growing in liquid SC media, and then harvested at log phase (5hr), 24hr, or 48 hr. **(E)** Quantitative CLS assay showing that lifespan extension in the *snf1*∆* cyc8-G337*S bypass mutant is fully dependent on *CAT8*. The strains are JS1256, WT; JS1394, *snf1*∆; JS1416, *snf1*∆* cyc8-G337S*; JS1506 *snf1*∆* cyc8-G337S cat8*∆; JS1509, *cat8*∆; JS1513, *snf1*∆* cat8*∆. Error bars in each experiment indicate standard deviation of the mean viability for 3 biological replicates. For qRT-PCR analysis, significant expression increases in the adapted mutants over the *snf1*∆ parent are indicated by an asterisk (p<0.05, one-tailed t-test).

Snf1 phosphorylates Mig1 under low glucose conditions to activate genes for alternative carbon source utilization 32. Among these targets is *CAT8*, which encodes a zinc cluster transcriptional activator of glyoxylate and gluconeogenesis pathway genes such as *ICL1*, *MLS1*, and *PCK1* (Fig. 6A) 50,51. The Cat8 protein is also directly activated by Snf1-dependent phosphorylation 52,53, and further transactivates its own gene as a positive feedback 54. *CAT8* is required for CR-mediated extension of CLS and for CR-amplified expression of select target genes during the di-auxic shift, including the acetyl CoA synthetase gene *ACS1* 19. Accordingly, *ACS1* transcription was partially restored in the *snf1*∆* cyc8-G337S* suppressor mutant (JS1416) compared to the *snf1*∆ mutant (JS1394) as measured through RNA-seq, as were numerous other genes that function in ethanol and acetate catabolism (Fig. 7A; Supplemental Tables S1 and S2). *CAT8* was among the significantly enriched transcription factors on the promoters of such genes (Supplemental Table S3; p = 1.58E-06), and qRT-PCR analysis confirmed its transcription was partially restored in JS1416 compared to JS1394 (Fig. 7B). Surprisingly, *CAT8* transcription levels modestly increased over time in the *snf1*∆ mutant, suggesting that additional Snf1-independent factors contribute to its activation. The key glyoxylate cycle gene *ICL1* (isocitrate lyase) was absent from the lists of genes with improved expression after 24 hr and 48 hr (Fig. 7A; Supplemental Tables S1 and S2), but we confirmed its expression was elevated using qRT-PCR (Fig. 7C). Unlike the *CAT8* gene, *ICL1* expression did not increase over time in the *snf1*∆ mutant (Fig. 7C), indicating it is normally more dependent on Snf1 for activation. Consistent with a partial restoration of glyoxylate cycle gene expression, trehalose accumulation driven by gluconeogenesis was absent from the *snf1*∆ mutant at 24 and 48 hr, but noticeably recovered in the *cyc8-G337S* suppressor (Fig. 7D). This is significant because trehalose accumulation during the post-diauxic shift period (~24 hr in our system) was previously linked with CLS extension by CR 55, and glyoxylate/gluconeogenesis flux to trehalose appears to favor quiescence more so than mitochondrial respiration 56,57.

Since *CAT8* expression was restored in the adapted mutants and *CAT8* was required for CLS extension by CR 19, we hypothesized that longevity in the adapted *snf1*∆ mutants was also mediated by *CAT8*. Importantly, deleting *CAT8* by itself has little effect on CLS in non-restricted SC medium (19 and Fig. 7E, JS1509), perhaps due to redundancy with other factors such as Adr1 and Sip4. However, deleting *CAT8* from the JS1416 (*snf1*∆* cyc8-G337S*) isolate completely blocked longevity of the resulting *snf1*∆* cyc8-G337S cat8*∆ triple mutant (JS1506) when grown in non-restricted SC media (Fig. 7E). We therefore conclude that the *cyc8-G337S* mutation isolated under CR growth conditions is compensating for a critical long-term survival function normally carried out by Snf1 during CR, namely the relief of transcriptional glucose repression. Limited CLS recovery and partially restored expression of numerous non-Mig1 target genes in the suppressors also indicate that additional Snf1-driven processes significantly contribute to achieving maximal CLS in normal cells under non-restricted conditions.

## DISCUSSION

SNF1 clearly supports cell survival when culture medium is depleted for glucose, the highly preferred carbon source of budding yeast. Cells lacking the Snf1 α-subunit or Snf4 γ-subunit fail to even grow when solely provided with less favorable carbon sources such as ethanol, acetate, glycerol, or sucrose 2. The *SNF* gene name derives from Sucrose Non-Fermenting because such genes are required for expression of *SUC2*, which encodes the sucrose hydrolyzing enzyme invertase 37,58. Suppressor mutations were originally identified in *snf1* mutant strains by isolating re-vertant colonies that grew on sucrose-containing or other types of alternative carbon sources 38,39. This is how the *SSN* genes were identified, including *CYC8/SSN6*. In the current study we identified adaptations that extended CLS of a *snf1*∆ parental strain, rather than restoring exponential growth on an alternative carbon source. In addition to relaxing glucose repression of gene expression, SNF1 also directly or indirectly regulates other transcription factors, kinases, and metabolic enzymes 59. Given such diversity in SNF1 targets, we anticipated that multiple cellular processes would be identified from the screen, including stress resistance pathways, metabolism, and transcription. The idea was to find the key processes that drive Snf1-dependent longevity. It was therefore quite remarkable and telling that the adaptive mutations were so specific to *CYC8* and *MIG1*. This clearly implicated the Mig1-mediated glucose repression system in CLS and CR, but did not rule out contributions from other downstream Snf1 targets (direct or indirect), especially since the suppressive effect of *cyc8* and *mig1* mutations was partial. Indeed, RNA-seq analysis for one of the adapted mutants (JS1416) indicated numerous Snf1-dependent genes whose transcription was at least partially restored by the *cyc8 G337S* mutation. Many of these genes harbored Msn2/4, Gis1, or Rph1 bind-ing sites in their promoters, which is significant because overexpression of these transcription factors suppresses phenotypic defects of *snf1* mutants 47,48. We therefore hypothesize that a combination of Snf1-driven processes are required for maximal CLS, not just relief of glucose repression. It is possible that other types of suppressor mutations would be identified with additional screening for adaptive mutants, or transcriptional relief of glucose repression is the only suppressible Snf1-dependent process that supports entry into quiescence, a property important for stationary phase survival.

Cyc8/Tup1 is a general transcriptional co-repressor complex that interacts with several different repressors of various cellular processes, including Mig1 (glucose repression), MATα2 (mating type gene regulation), Rfx1 (DNA damage regulated genes), and Sko1 (osmotic and oxidative stress response) 31. Interestingly, a common theme for some of these factors is that they not only function as transcriptional repressors, but also contribute to activation under specific conditions 35,60,61. For example, under activating conditions like growth on galactose or ethanol, Cyc8/Tup1 forms a complex with Cti6 and phosphoinositol (3,5) bisphosphate to function as a co-activator with Mig1, aiding in recruitment of the SAGA histone acetyltransferase complex to affected genes 45,62,63. Cyc8/Tup1 is thought to mask the activation domain of Mig1 through physical interaction 64. Phosphorylation of Mig1 by SNF1 in response to low glucose then alters the interaction such that its activation domain is unmasked 35,64. With Mig1 and Cyc8/Tup1 being important for the Snf1-mediated transition from glycolysis/fermentation to respiration and gluconeogenesis, it is likely the adaptive Cyc8 mutations identified as CLS bypass suppressors disrupt masking by Cyc8/Tup1 while still allowing for transcriptional activation. This is consistent with the large number of genes in the JS1416 suppressor strain that are super-activated compared to *snf1*∆ or even the WT strain (Supplemental Tables S1 and S3). It is not clear whether specific TPR motifs or other domains of Cyc8 are required for interaction with the SAGA complex, though phosphoinositol (3,5) bisphosphate and Cti6 direct the transition from co-repressor to co-activator 45,63.

Some of the adapted *snf1*∆ mutants have mutations in *CYC8* and one or more additional genes. For example, A10-1 and A10-2 both have the same *CYC8* mutation (W367L) and are probably sibs for that allele, but A10-2 also has mutations in *CKS1* and *AIM24* (Table 1). In this case, we hypothesize that the *cyc8-W367L* mutation occurred early on within the culture, followed by *cks1* and *aim24* mutations arising in a subsequent daughter cell. There are similar examples of sequential mutations from other cultures (Table 1; A1, A3, A4, A9), suggesting that the adapted *snf1*∆ mutants remain mutagenic. It is possible that certain secondary mutations could further modify CLS of the adapted strains such that they gain a more competitive regrowth/survival advantage, but they are not expected to significantly impact CLS on their own because they all accompany *cyc8* mutations and were never observed more than once.

### Cyc8 as an adaptable hub for transcriptionally regulating carbon metabolism

Cyc8 as a key mediator of Snf1 function during chronological aging is especially intriguing because it contains glutamine-rich repeats (Fig. 5A) than confer the ability to form a prion known as [*OCT*^+^], a heritable amyloid-like aggregate in the cytoplasm 65. [*OCT*^+^] as an aggregated Cyc8 protein becomes inactive and results in phenotypes similar to deleting *CYC8*, such as derepression of Cyc8/Tup1 repressed genes and flocculence 65. Interestingly, the polyQ/N repeats of Cyc8 are variable between naturally occurring yeast strains, with longer repeats resulting in higher expression of Cyc8/Tup1 target genes 66. Mutations in the glutamine-rich repeats were not identified in the adapted *snf1*∆ mutants, consistent with the absence of non-sense mutations that completely blocked Cyc8 function. Though it is currently unknown if [*OCT*^+^] is a CLS determinant, the frequency of [*PSI*^+^] prion formation is increased with chronological aging 67, and prion formation is also triggered by oxidative stress 68. Perhaps [*OCT*^+^] is also triggered by oxidative stress, especially in a *snf1*∆ mutant. One intriguing possibility is that the TPR9 and TPR10 adaptive mutations of *CYC8* protect against prion formation by promoting new physical interactions with drivers of transcription that stabilize the protein or alter association with Tup1. Given that a simple point mutation in the TPR9 repeat of Cyc8 in JS1416 shortens CLS independently of *SNF1* (Fig. 5C), modulation of Cyc8 polyQ/N repeat length and aggregation could also naturally impact cell survival during quiescence.

### The utility of adaptive regrowth as a screen for chronological aging factors

Chronological aging appears especially suited to the idea of altruistic aging, whereby programmed cell death (apoptosis) within a population of unicellular organisms benefits the survival of individual cells that are better adapted to the environment, usually due to spontaneous mutations 26. This idea is controversial and difficult to reconcile with the more complex biology of multicellular organisms 69. We tend to view this phenomenon in yeast as an extreme form of accelerated evolutionary selection. Cell survival in stationary phase cultures puts a strong selective pressure on the population, especially given the high ROS levels in some short-lived mutants such as *snf1*∆. Evidence for continual adaptation and selection within the cultures comes from the fact that we typically had to stop the quantitative CLS assays at ~14 days because the adapted *snf1*∆ strains that obtained *cyc8* mutations would then undergo a new round of adaptation as they were chronologically aged, resulting in “gasping” again that strongly suggests selection for additional mutations. With the accessibility of multiplexed whole-genome sequencing and the small genome size of *S. cerevisiae*, using adaptive regrowth to identify mutations that restore longevity to short-lived mutants (such as *snf1*∆) is a powerful modern spin on the "old school" method of suppressor screening being applied to the problem of aging.

## MATERIALS AND METHODS

### Yeast strains, plasmids, and media

All yeast strains in this study were derived from the BY4741/BY4742 background 70 either through direct transformation or genetic crossing/tetrad dissection. Gene deletions were constructed by one-step replacement with *kanMX4* PCR products, and then correct integration was confirmed by PCR from genomic DNA or colony PCR. To avoid potential mutation accumulation that could occur over time in the KO collection, we deleted and replaced one allele of *SNF1* from BY4743 to generate JS1230 and then dissected tetrads. The "WT" strain JS1256 was derived from this cross and has the same genotype as the *MAT*a KO collection strain BY4741. JS1394 and JS1399 *snf1*∆ haploid strains were derived from the same cross. Diploid strains E1 through E20 were generated for dominance tests by crossing JS1399 with each of the suppressor mutants (JS1413 - JS1432). We could not confirm the *CYC8* deletion in the original knockout collection strain, so it was remade in JS1256 and then crossed to JS1399 to generate a heterozygous diploid JS1475. This strain was sporulated and tetrads dissected to obtain *MAT*a *cyc8*∆ (JS1490) and *cyc8*∆* snf1*∆ (JS1491) haploid strains. Genotypes are provided in Table S4. The *SNF1*-containing plasmid pSNF1-316 was kindly provided by Martin Schmidt 71. Where indicated, strains were grown in rich Yeast Extract/Peptone Dextrose (YPD) or Synthetic Complete (SC) medium containing either 2% (NR) or 0.5% (CR) glucose 18. All cell growth was at 30°C.

### ROS and mutation frequency assays

BY4741 cell cultures were started at OD_600_ of 0.015 in SC medium containing either 2% or 0.5% glucose. At time points of 9, 24, and 48 hours, cell aliquots were harvested, washed with incubation buffer (10 mM MES, 100 mM KCl, 140 mM NaCl, pH6.5), and then 1x10^7^ cells were resuspended in 1 ml of incubation buffer. Dihydrorhodamine 123 (1 mg/ml) was added to each reaction (1x10^7^ cells/ml) to obtain a final concentration of 5 µM. Cells were incubated for 10 minutes at room temperature, and then fluorescence intensity was measured using a Spectramax plate reader at excitation 504 nm / emission 534 nm. For each condition, three biological replicates were tested and normalized to the mean signal of log phase WT NR (2% glucose) samples at a value of 1.0.

Spontaneous mutation of the *CAN1* gene was detected by colony formation on SC medium lacking arginine and supplemented with 60 µg/ml canavanine (SC-arg+can). BY4741 was grown overnight in SC 2% glucose and then inoculated to a starting OD_600_ of 0.015 and allowed to grow into log phase (~0.5 OD_600_), the late diauxic shift (24 hr), and the early stage of stationary phase (48 hr). At each time point, cells were spread directly onto SC-arg+can plates or serially diluted with sterile water so that between 200 and 300 cells were being spread onto SC plates. Relative frequency of canavanine resistance was calculated by the ratio of canavanine-resistant colonies to total colonies on SC. The experiment was performed in biological triplicate for each condition, with three technical replicates.

### Genetic screen for *snf1*∆ bypass suppressors of short CLS

The *snf1*∆*::kanMX4* haploid strain JS1394 was streaked onto a YPD plate for single colonies from a frozen stock. One colony was then restreaked again onto YPD and 10 independent colonies (A1 through A10) were inoculated into 5 ml of SC 2% glucose media in 15 ml glass tubes with loose fitting metal caps and grown overnight while rotating in a New Brunswick Scientific roller drum at 30°C. Next, 20 µl of the overnight cultures were inoculated into 10 ml of fresh SC 2% and SC 0.5% cultures, and then incubated in the roller drum. Every 2-3 days, 20 µl aliquots were 10-fold serially diluted into sterile water, spotted onto YPD 2% agar plates, incubated for 2 days of colony growth, and photographed as previously described for qualitative CLS assays 15. Starting at day 13 and continuing through day 17, candidate bypass suppressor colonies were picked from the YPD plates as they appeared, single colony purified, and permanently frozen as JS1413 through JS1432. Spot test growth assays on alternative carbon sources was performed by patching each strain onto a YPD plate, which was then grown overnight. The following day, cells were scraped from the patches and resuspended in 1ml of sterile water. Each sample was then normalized to an OD_600_ of 1.0 and 10-fold serially diluted in a 96-well plate, followed by spotting 2.5 µl onto YEP plates containing either 2% glucose, 2% ethanol/2% glycerol, or 2% potassium acetate, and incubated at 30°C. Photos were taken at day 2, 5, and 6.

### Whole genome sequencing library preparation and analysis

Individual suppressor mutants were grown overnight in 5 ml of YPD. A YeaStar (Zymo Research) DNA kit was then used to purify genomic DNA for 1.5 ml of cells in 3 different spin columns. DNA was eluted in 60 µl of dH_2_O for each spin column and pooled for a total volume of 180 µl per strain. DNA was then sheared using a Diagenode Bioruptor for 45 cycles of 30 seconds "ON" and "OFF" per cycle. Sheared DNA was quantified using a Qubit fluorometer (Invitrogen) and a minimum of 1µg was used as input for NEBNext DNA library construction kit (E6040S). Libraries were prepared according to NEB guidelines and quality controlled with Agilent High Sensitivity DNA chips (5067-4626) for library size and concentration. Finalized libraries were multiplexed and sequenced on an Illumina Miseq at the UVA Genome Analysis & Technology Core Facility. Sequencing reads were mapped to the sacCer3 genome assembly using bowtie2 72. Samtools suite v.1.2 was used to sort and filter duplicates from the resulting bam files 73. Genotype calls were then made with samtools mpileup while variants were called with bcftools call v.1.2. The final variant call files were modified to make them compatible with the IGV genome browser. FASTQ files for the whole genome sequencing were deposited into NCBI BioProject as accession# PRJNA396952.

### Quantitative CLS assays

The CLS assays were performed as previously described 19. Three single colonies of each strain were inoculated into 10 ml of SC or SC-ura media containing 2% glucose, then grown overnight while rotating in a roller drum at 30°C. A 100 µl aliquot of the overnight culture was used to inoculate a fresh SC or SC-ura culture containing either 2% or 0.5% glucose. Starting at day 2 (for SC) or day 4 (for SC-ura) 20 µl aliquots were removed and serially diluted 10-fold. 2.5 µl of the 1:10, 1:100, and 1:1000 dilutions were spotted onto YPD or SC-ura plates (for strains harboring a *URA3* vector) such that 12 cultures are tested on each plate and incubated at 30°C. Photos of microcolonies growing within each spot are taken on a Nikon Eclipse 400 tetrad dissection microscope and the number of colonies counted. The fraction of cells viable at each day compared to the first day of testing (day 2 or 4) was calculated for subsequent days.

### RT-PCR assays

JS1256, JS1394, JS1416, and JS1417 were inoculated from single colonies into 10 ml SC (2% glucose) cultures and grown overnight on the roller drum at 30°C. Fresh cultures were then inoculated to an OD_600_ of 0.02 in 100 ml SC (2% glucose) in 250 ml Erlenmeyer flasks. RNA isolation and quantitative RT-PCR was then performed for the *CAT8*, *ICL1*, and *TFC1* genes as previously described 16. Briefly, aliquots of the cultures were harvested at the indicated time points and total RNA isolated by the acidic phenol method 74, and then converted into cDNA using a Verso cDNA Synthesis Kit (Thermo Scientific) according to the manufacturer instructions. Quantitative PCR was then performed using SensiMix SYBR Hi-ROX Mastermix (Bioline) on an Applied Biosystems Step-One Plus real-time PCR instrument as previously described 16. Relative expression level for each tested gene was calculated by normalized to the control *TFC1* RNA level 16. Three biological replicates were run for each strain. The primer sequences are listed in Table 2.

**Table 2:** RT-PCR primer sequences.

**Primer**	**Gene**	**Sequence**
JS2630	*CAT8 *FW	GCATGGAATGGACAACTGTG
JS2631	*CAT8 *REV	GACCGATCCAAGTCATCGTT
JS2389	*ICL1 *FW	GCATTTGCTCCATATGCTGA
JS2390	*ICL1 *REV	TTTGGCCAGTTAAAGGATGG
JS2399	*ACS1 *FW	TGATGACGCGCTAAGAGAGA
JS2400	*ACS1 *REV	AACGGGTGTGCATGGATAGT
JS2022	*TFC1 *FW	AATGTACCAAAGCCACCACCC
JS2023	*TFC1 *REV	ACCGCCTGGAGTGTCTGATT

### Trehalose measurements

Yeast cultures were grown overnight in a 10 ml SC (2% glucose) culture and then re-inoculated into 50 ml SC medium (2% glucose) to A600=0.2. The cultures were then incubated shaking in a water bath. Aliquots were harvested after 5 (log phase), 24, and 48 hr, and the H_2_O-washed cell pellets frozen at -80°C. Trehalose was extracted from pellets (~20 mg wet weight) using the method of Parrou and Francois, which utilizes trehalase (Sigma) to convert trehalose to glucose 75. The released glucose was then measured using a colorimetric glucose measurement kit (Sigma) with a glucose standard curve.

### RNA-seq library construction and data analysis

Triplicate cell cultures were incubated in 10 ml of SC medium containing 2% glucose, rotating at 30°C in a roller drum. 2 ml aliquots were harvested after 6 hr (log phase) and 24 hr of growth, and the cell pellets were collected after 2 rounds of centrifugation and washing with ice cold water. RNA was then isolated from the cell pellets using the hot acid phenol method and resuspended in 50 µl of DEPC treated water 73. Following treatment with DNase I for 15 min at 37°C, RNA concentrations were quantified using a Nanodrop 2000c spectrophotometer (Thermo Scientific). Libraries were constructed using NEBNext Poly(A) mRNA magnetic isolation (E7490) and NEBNext Ultra Directional II (E7760) kits with 3 µg of RNA as input. Kits were used according to manufacturer instructions, except the libraries were sized selected between 250-500 bp on a 1.5% agarose gel and gel purified using a Zymogen gel purification kit. The barcoded libraries were sequenced (75 bp single end reads) on an Illumina Nextseq 500 at the UVA Genome Analysis & Technology Core facility. Sequencing data is available at GEO (accession number GSE110409)

The fastq files were combined from separate lanes and mapped to the sacCer3 reference genome using bowtie2 with default options. A read counts file was obtained using a combination of bedtools multicov and a genome bed file containing all yeast ORFs. The resulting counts file was analyzed with the edgeR software package to produce a list of differentially expressed genes for all samples at 24 and 48 hours compared to log phase of the respective samples with a false discovery rate (FDR) <0.05 76. The list was further filtered to only include genes with reduced expression in the *snf1*∆ mutant compared to WT. Finally, a heatmap was produced from this list of 986 genes using the Pearson correlation clustering method with pheatmap v.0.7.7 (http://CRAN.R-project.org/package=pheatmap . Gene clusters were then examined for significant gene ontology (GO) term enrichment using Yeastmine in the *Saccharomyces *Genome Database 77.

## SUPPLEMENTAL MATERIAL

Click here for supplemental data file.

All supplemental data for this article are also available online at http://microbialcell.com/researcharticles/spontaneous-mutations-in-cyc8-and-mig1-suppress-the-short-chronological-lifespan-of-budding-yeast-lacking-snf1-ampk/.
